# Integrated microwave cavity perturbation sensor for nondestructive estimation of moisture content of grains: A case study on wheat and chickpea

**DOI:** 10.1371/journal.pone.0343435

**Published:** 2026-03-05

**Authors:** Kamil Sacilik, Necati Cetin, Burak Ozbey, Fernando Auat Cheein

**Affiliations:** 1 Department of Agricultural Machinery and Technologies Engineering, Faculty of Agriculture, Ankara University, Ankara, Türkiye; 2 Department of Electrical and Electronics Engineering, Faculty of Engineering, Ankara University, Ankara, Türkiye; 3 School of Mathematics and Computer Science, Heriot Watt University, Scotland, United Kingdom; 4 Department of Engineering, Harper Adams University, Newport, England, United Kingdom; Central Food Technological Research Institute CSIR, INDIA

## Abstract

This study presents a low-cost, integrated microwave cavity perturbation sensor operating in TM_010_ mode at 2.45 GHz for the non-destructive estimation of moisture content (MC) in wheat and chickpea grains. Unlike conventional methods that rely on expensive vector network analyzers (VNAs), this system uses a custom-designed circuit to derive a density-independent moisture content function, M(*Ψ*). Various machine learning models (ML) were trained to predict MC and classify grain types based on these dialectical metrics. The results demonstrate that bagging (BAG), k-nearest neighbors (k-NN), and reduced-error pruning trees (REPTree) models significantly outperform deep learning models. The BAG model achieved the highest predictive performance, yielding correlation coefficients (R) of 0.995 for wheat and 0.989 for chickpea, with root mean square error (RMSE) of 0.207% and 0.302%, respectively. Furthermore, k-NN variants achieved 100% accuracy in classifying the grain types. The proposed system offers a precise, rapid, and cost-effective alternative for real-time grain quality monitoring.

## 1. Introduction

Wheat and chickpea are two important crops worldwide, contributing to food security and nutritional quality. Wheat (*Triticum aestivum* L.) is a staple cereal that serves as a major source of carbohydrates and energy, forming a crucial part of daily caloric intake in many diets [1]. Chickpea (*Cicer arietinum* L.), a leguminous crop, provides protein, fibre, and essential nutrients, complementing wheat nutritionally [2,3]. Additionally, incorporating chickpea flour into wheat-based products enhances their nutritional value by increasing protein and essential fatty acid content while also reducing environmental impact [4].

The moisture content (MC) of wheat and chickpea grains plays a critical role in determining their quality, storage, and processing, necessitating accurate characterisation in the food grain industry. MC significantly influences the physical, chemical, and physiological properties of these grains, as well as their susceptibility to microbial growth and spoilage. Precise moisture measurement is essential for successful harvesting and post-harvest operations, capacity planning, and pricing in the grain trade [5–7]. The physical properties of grains are directly affected by MC, which in turn influences the design and operation of equipment used for harvesting, handling, and processing [8]. Maintaining optimal moisture levels is crucial for preventing spoilage and preserving both the nutritional and economic value of stored grains [9].

MC also plays a key role in mycotoxin formation during cereal storage and processing, as high moisture levels promote fungal growth and mycotoxin production [10]. Mycotoxins in wheat pose a danger to human health, as they have been shown to be potentially carcinogenic [11]. The interaction between grains and biological factors is influenced by MC, thereby affecting post-harvest losses and, ultimately, the nutritional and economic value of grains [8]. Consequently, accurate MC determination is vital for grain handling, storage, and transportation processes [12]. It also affects the biophysical properties of grains, helping to prevent storage and processing issues [13]. Furthermore, MC is a critical factor in drying and milling operations, as variations in chickpea MC, for example, influence milling parameters and flour composition [14]. The storage potential and quality of wheat are directly linked to MC at harvest and storage. Wheat harvested with an MC above 13% is prone to fungal contamination and a reduction in both physical and physiological quality [15]. Maintaining optimal MC is, therefore, essential for preserving grain quality, nutritional value, and functionality, all of which are fundamental to grain storage, processing, utilisation, food security, and agricultural practices. Proper MC characterisation and control are critical for optimising the entire grain supply chain from production to market.

Advanced techniques, such as microwave sensing and permittivity measurement, have enabled rapid and non-destructive MC determination, ensuring effective grain quality management and improved processing conditions [16,17]. The integration of machine learning (ML) with these sensing technologies has enhanced the accuracy and efficiency of MC estimation. Microwave measurement techniques combined with ML can analyse complex data to provide highly precise moisture predictions. Traditional MC measurement methods, however, are often time-consuming and labour-intensive, as manual sampling and analysis may lead to uneven moisture assessments within storage facilities [18]. Non-destructive testing methods, such as microwave characterisation and ML, enable real-time MC monitoring without damaging the grain, which is crucial for maintaining quality during storage and processing [19].

Dielectric properties and microwave signal characteristics have been successfully employed to improve MC prediction accuracy using ML models, including random forest, support vector machines (SVM), and artificial neural networks (ANN) [18,20,21]. These methods can process data from various microwave frequencies and environmental conditions, providing real-time, non-invasive, and reliable moisture measurements [20–22]. The use of SVM in conjunction with dielectric characteristics has demonstrated highly accurate wheat MC prediction, achieving an R² of 0.998 [19]. Additionally, ANN models trained on dielectric data from RF transceivers have achieved over 98% accuracy in predicting rice MC using multiple input features [18]. By capturing the non-linear relationships between dielectric properties and MC, these models enable precise moisture estimation in the complex biological structures of grains [17].

Machine learning integrated with dielectric measurement techniques enhances the accuracy of MC predictions and facilitates real-time monitoring, which is essential for maintaining optimal storage conditions and preventing spoilage [23]. The integration of ML with microwave sensing systems significantly improves MC estimation precision while promoting the development of portable, cost-effective, and user-friendly devices for agricultural applications [21,22]. This approach is particularly valuable for achieving accurate moisture estimation in situations where conventional MC determination methods are labour-intensive and time-consuming.

Unlike previous studies that utilize ML solely for data-driven predictions without physical context, this study integrates artificial intelligence (AI) with a custom-designed microwave cavity perturbation sensor governed by electromagnetic theory. While the derived metric M(Ψ) theoretically mitigates bulk density effects, the relationship between this parameter and moisture content is not strictly linear due to the complex biological structure of the grains and deviations from ideal perturbation conditions (e.g., sample shape irregularities and volume variations). Classical linear regression models often fail to capture these subtle, non-linear dependencies. Therefore, the primary rationale for employing machine learning in this work is to utilize it as a robust, non-linear calibration tool that bridges the gap between theoretical microwave physics and real-world experimental variability, thereby enhancing estimation accuracy beyond the limits of traditional analytical methods. While conventional systems rely on expensive VNAs, our study introduces a physically grounded metric (M(Ψ)) for moisture estimation, contributing a novel approach to measurement science. Conventional moisture determination methods often involve time-consuming, destructive procedures with limited accuracy per sample. In this study, the primary rationale for using machine learning algorithms is to effectively model the complex and non-linear relationship between moisture content and the function M(Ψ), a physically meaningful parameter derived from microwave resonance measurements. Machine learning can learn such relationships with high accuracy, where classical regression methods struggle to resolve them. In this study, machine learning is integrated with physical measurement principles. The resulting models are both physically meaningful and provide measurement-based reliability. This approach addresses the need for rapid, non-destructive, and cost-effective moisture determination of granular agricultural products.

The electrical properties of materials can be used effectively to characterize many natural phenomena. Since the complex permittivity $\varepsilon^{*}$ is a function of many physical stimuli such as pressure, strain, humidity, etc. It is possible to design a sensing system based on observing a change in permittivity induced by a stimulus. The measurement systems for the dielectric properties of materials are advanced, highly sensitive, and non-hazardous to human health. The moisture content (MC) of a material also modifies its dielectric properties, which implies that a moisture sensor based on the change in $\varepsilon^{*}\;$ is not only theoretically possible but is also the preferred one thanks to these qualities.

Specifically, utilization of radio frequency and microwave dielectric characterization methods for measuring the MC of grains has been extensively demonstrated in the literature. These methods can be classified into resonance-based and non-resonance-based methods. The first group of techniques, such as the microwave resonant cavity, accounts for the variation in the microwave system's resonance frequency, which typically shifts when the MC of the sample under test is modified. The second group includes techniques that rely on the sample's reflection and transmission properties, such as free-space methods [24,25]. Typically, resonance-based methods yield a better accuracy and superior sensitivity compared to non-resonance methods [26]. On the other hand, free-space methods enable wireless characterization in addition to non-destructive measurements, however, at the expense of sensitivity, resolution, and accuracy. Since the system can only be isolated to a certain degree, typically, reflections from the clutter contaminate the frequency spectrum, resulting in reduced accuracy. In addition, attenuation variation is more difficult to characterize than resonance-frequency variation in resonance-based methods.

Resonance-based methods are a better alternative for handling some of these problems. These methods rely on variations in resonance characteristics, such as shifts in peaks or dips in the reflection or transmission spectra of samples under test with MC. In [27] and [28], stray- or fringing-field resonator designs based on the microwave resonance technique were demonstrated to perform density-independent measurements of MC for pharmaceutical products. Similarly, in [29], a microstrip-ring-resonator-based microwave system was designed to measure the MC of microcrystalline cellulose and maize starch. However, by far the most popular resonance-based characterization method is the cavity perturbation technique [30]. This technique offers high sensitivity and resolution, primarily because of its high quality factor (Q). Q can be defined as the ratio of the average stored energy to the lost energy per second in a resonator [31]. Therefore, a higher Q value can be achieved by reducing losses. In a cavity resonator, this is enabled by its completely metal structure, which allows only certain waveguide modes to propagate, determined by the shape and feeding mechanism. The cavity perturbation technique utilizes changes in the resonance frequency and quality factor to extract the complex permittivity$\varepsilon^{*}$. An increase in the water content of the sample under test inside the cavity leads to an increase in the magnitude of $\varepsilon^{*}$, which in turn decreases the resonance frequency and Q. Hence, the measurement of the frequency shift and quality factor enables high-sensitivity, high-accuracy, and non-destructive measurement of agricultural products, both as single kernels or granules. The cavity perturbation technique has been used to detect the MC of single wheat grain kernels [32,33], single rice grains [33], cellulose and corn granules [34], low MC food products such as corn starch, curry, and paprika [33], in addition to the MC of various other organic and inorganic materials [35–38].

A resonant cavity is a type of microwave resonator constructed by short-circuiting a waveguide at both ends. This results in a closed-box structure, which can trap electromagnetic waves with high Q. The cavity perturbation technique characterizes the change in the electromagnetic properties of a cavity resonator when a sample is introduced into it. The measurement can either be performed by introducing a slot in the structure through which the sample is placed [38–40], or by placing the sample directly inside the cavity [41]. The first method does not require the cavity to be opened up and closed each time the sample is placed, while in the second method, it is necessary. However, the second method has the advantage of preserving the classical shape of the cavity, which is generally designed in rectangular or cylindrical form, as this may affect its resonance characteristics.

In most studies that demonstrated permittivity estimation using cavity perturbation, a vector network analyzer (VNA) is employed to measure $S_{\text{2}\text{1}}$. A VNA is a device that can characterize a microwave network over a wide frequency range and is used to obtain the complex transmission/reflection parameters. A vast majority of state-of-the-art agricultural sensing/detection platforms based on microwave techniques use either benchtop or handheld VNAs [17,34,42,43]. Although it is standard equipment for RF and microwave measurements, its high cost and limited mobility, aside from some handheld models, make it undesirable for practical applications and confine its use to the laboratory environment. Therefore, a compact device that can replace the VNA in performing $S_{\text{2}\text{1}}$ measurements of the cavity resonator is invaluable for reducing costs and enhancing the practicality of microwave MC measurements.

In this work, a cylindrical cavity resonator operating in the TM_010_ mode is designed at 2.45 GHz, and it is demonstrated that it can be used for high-sensitivity, high-accuracy MC characterization of granular agricultural products. Furthermore, to replace the high-cost VNA, a full microwave measurement system is designed and fabricated. The system, based on I/Q data, is shown to be capable of performing complex $S_{\text{2}\text{1}}$ measurements and can be integrated with a computer for data processing. Experimental data acquired via numerous trials of perturbed cavity measurements with many wheat and chickpea grains in single kernel forms are then employed in machine learning models to characterize the relationship between parameter X and MC. Therefore, the system proposed in this study enables low-cost and ultrasensitive MC estimation of two of the most essential agricultural products.

## 2. Materials and methods

**Fig 1:**
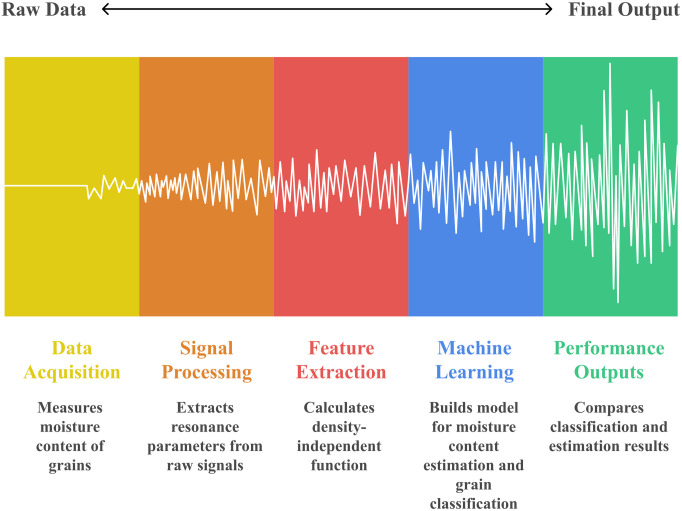
The flow chart of the study.

The measurement system was developed using a microwave resonator with a well-defined geometry. Resonance frequency and bandwidth values were recorded and digitized for post-processing. The M(Ψ) function was derived from these parameters and used as the target variable for ML model training. Hyperparameter tuning was conducted using 10-fold cross-validation. Parameter ranges and model configurations are provided in the modeling section, ensuring full reproducibility of the methodology. Fig 1 shows the five-stage data processing pipeline of the proposed system, from raw data acquisition to final performance output. The process includes acquiring microwave signals from grains, processing resonance parameters from raw signals, deriving the M(Ψ) function, training artificial intelligence models, and finally verifying the prediction and classification results.

### 2.1. Preparation of the samples

Wheat (*cv*. İkizce) and chickpea (*cv*. Akçin) samples used in this study were obtained from the Ankara Field Crops Central Research Institute, Türkiye. The seeds at reception were manually cleaned to remove foreign matter, immature, and broken seeds. The initial MC of the materials used in this study was determined with a halogen moisture meter (Mettler Toledo HB43-S, Schwerzenbach, Switzerland). The MC of the product in each experimental period was measured as % wet basis (% w.b.) using a halogen moisture meter. The initial MC of wheat and chickpea was 10.07 ± 1 % w.b. and 9.33 ± 1 %w.b., respectively. In order to determine the effect of MC on the moisture function of seeds, the wheat and chickpea samples were conditioned to obtain different MC levels in the range of 10.07–16.63% w.b., and 9.33–15.96% w.b., with an increase of about 0.57 w.b., respectively. Samples of desired moisture level within the above range were prepared by adding calculated amounts of distilled water and sealing them in separate plastic containers. The samples were kept in a refrigerator at 5 ºC for at least 1 week and stirred frequently by rotating the containers to ensure uniform moisture distribution throughout the samples. Before each test, the required quantity of seeds was taken out of the refrigerator and allowed to warm up to room temperature for 12 h. [44]. All experiments were carried out under laboratory conditions at 22 ± 0.5 °C. The wheat and chickpea samples were tested at 12 different moisture levels. At every measurement, the amount of the sample placed within the cavity was kept as close as possible (6–10 g of wheat and 3–4 chickpea kernels depending on the size). For each MC level, 50 measurements were performed by changing the samples under test. This enables the data acquired to be statistically meaningful and applicable to machine-learning methods.

### 2.2. The dielectric spectroscopy setup

**Fig 2:**
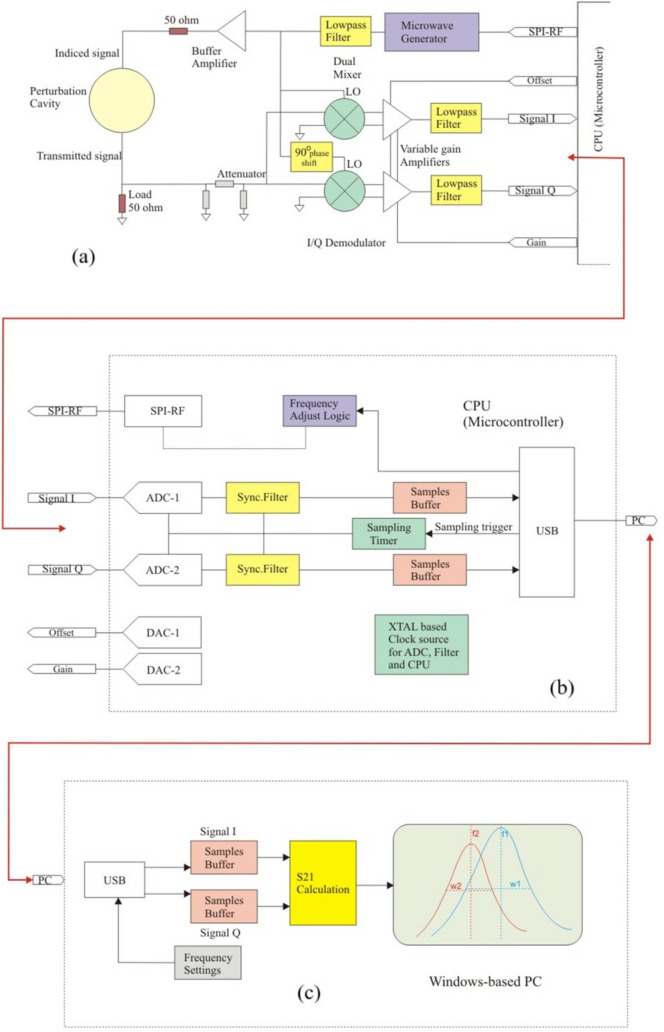
Microwave dielectric spectroscopy setup based on perturbed cavity resonator.

The microwave MC characterization system based on the perturbed cavity method is illustrated in Fig 2. The system's essential components are a microwave generator, an I/Q demodulator, a dual analog-to-digital converter (ADC), and a microcontroller unit (MCU). The microwave generator is made up of a voltage-controlled oscillator (VCO) and a phase-locked loop (PLL) and it is able to produce a microwave signal in the 0.7–3.5 GHz range with an output power of −4 from dBm to 4 dBm. The microwave signal was passed through a low-pass filter (LPF) and boosted by a 20 dB amplifier. It was then sent to the cavity's input port via a 50 Ω transmission line. The purpose of the amplifier is to compensate for the losses in the cavity and other parts of the microwave setup, whereas the filter is used to obtain purely monotonic signals free of harmonics.

The cavity was designed in a cylindrical form to operate in the TM_010_ mode. This mode leads to an electric field distribution with its maximum along the cylinder's central axis, whereas the tangential magnetic field is maximum at the cylindrical cavity walls. TM_010_ is an ideal mode for placing the sample at the center of the cavity, where the maximum MC sensitivity is obtained. The resonance frequency of the empty cavity $f_{\text{1}}=\;$2.45 GHz. The cavity was designed without a sensing channel; i.e., the lid had to be opened before the sample was placed, then closed. This procedure was repeated for every sample. This configuration minimizes deviation from the cylindrical form, enabling an easier design process, as theoretical calculations are more easily matched to measurement results than in a highly perturbed cavity. However, in order to obtain the most accurate results, the volume of the sample must be much smaller (around 1/1000^th^) compared to the cavity volume in this geometry.

The cavity was excited through two holes onto which the SMA connectors were mounted, which were in turn connected by RF cables to the microwave generator. The rationale behind using these two ports is to investigate the transmission spectrum, expressed as $S_{\text{2}\text{1}}$ (in dB). Based on the measurements of $\left|S_{\text{2}\text{1}}\right|$, the magnitude of the complex transmission parameter $S_{\text{2}\text{1}}$ over a range of microwave frequencies, the resonance frequency and the bandwidth can be obtained when the cavity is empty and when it is filled with a volume of sample.

A microwave mixer was used to combine the cavity output signal with the unamplified signal from the microwave generator. The cavity output signal was also mixed with the original signal, with a 90° phase shift. The result of the first operation produces the DC in-phase signal (I), whereas the second mixer yields the DC quadrature (Q) signal. The I and Q signals do not contain any intermediate frequencies, since the mixer input is composed of two components at the same carrier frequency. Two LPFs are employed at the mixer output to eliminate parasitic frequencies introduced by mixing. The I and Q signals are fed into the ADCs, which digitize them with 16-bit resolution. The output signal is subjected to further digital synchronous filtering. Digital signal processing is carried out by an MCU, which is a fast microcomputer with a 32-bit ARM architecture with sufficient memory and floating-point DSP capabilities. The intensity of the cavity output signal is calculated from the measured I and Q signals, and this information is used to extract |S21| at every frequency within the band, yielding the cavity transmission spectrum. This in turn is employed to obtain the parameters $f_{\text{1}}$, $f_{\text{2}}$, $w_{\text{1}}$, and $w_{\text{2}}$.

### 2.3. Machine learning modelling

MC was predicted from the moisture content functions (M(Ψ)) of wheat and chickpea seeds separately using WEKA [45]. A procedure was utilized to create the different MC conditions. A total of 1200 samples (600 of each species) were analyzed without feature selection. First, *M*(*Ψ*) in the VNIR region was characterized. For each species, the inputs represent 600 different *M*(*Ψ*) values, and the outputs represent the MC values of the products. 10 different machine learning algorithms (random forest (RF), bagging (BAG), k-nearest neighbors (k-NN), deep learning (DL), Gaussian processes (GP), light gradient boosting machine (LGBM), multiple linear regression (MLR), multilayer perceptron (MLP), support vector regression (SVR), and reduced error pruning tree (REPTree)) were used for prediction. In addition, some regression models were removed, and the following models were added for classifying the wheat and chickpea: linear discriminant analysis (LDA), logistic regression (LR), support vector machine (SVM), Wilkie, Stonham and Aleksander Recognition Device (Wisard), Bayesian network (BayesNet), logistic model tree (LMT), decision tree (DT), and simple classification and regression tree (SimpleCART). The k-fold cross-validation method was used to create each model. In this study, the k-fold value was set to 10. This method divided the dataset into 10 equal sections, with a 9:1 split for the training and test sets. The learning procedure was carried out, with nine parts serving as training sets and one new part serving as the test set [46,47]. Validation was performed on the collected dataset using only this internal cross-validation procedure. Therefore, two different types (wheat and chickpea) were included in the study to demonstrate the models’ generalization capabilities. The learning procedure was performed by using nine sections as training sets and one new section as the test set.

The Poly kernel function was selected in the SVR and GP algorithms. In the MLP, the number of neurons in the hidden layer was twice the number of inputs in each model. A sigmoid activation function was used, the number of epochs was 500, the momentum coefficient was 0.1, and the learning ratio was 0.3. In the k-NN, k values of 1, 3, and 5 were chosen, and the Euclidean distance rule was chosen in the search process. In deep learning, the activation function is changed to sigmoid, tanH, and ReLu for DL-1, DL-2, and DL-3, respectively. In addition, the instance iterator, network configuration, iteration listener, zoo model, and loss function were selected as the default, neural network configuration, epoch, CustomNet, and Loss MSE, respectively. For LGBM, the objectives were binary (logistic) and multi-class (softmax). The maximum tree depth and minimum total weight of the instances in a leaf for REPTree were −1 and 2.0, respectively. The classifier for BAG was chosen as REPTree, and the number of iterations was 10. In the MLR, the ridge value was 1.0E-8, and the attribute selection method was the M5 method. The search algorithm and estimator for BayesNet were selected as K2 and the simple estimators, respectively. In the LDA and LR algorithms, the ridge values were chosen to be 1.0E-6 and 1.0E-8, respectively. The map type for Wisard was random. The DT's confidence factor was 0.25. In the LMT, the number of iterations for LogitBoost was −1. The number of folds in the internal cross-validation for SimpleCART was 5 (default). Approximately 600 MC and 600 *M*(*Ψ*) data points were utilized for each species (a total of 1200).

### 2.4. Determination of moisture content function

Microwave resonance technology (MRT) determines moisture content by measuring the interaction between water molecules and electromagnetic stray fields detected on the MRT sensor surfaces. This interaction modifies the electromagnetic field, and the resulting changes are measured [29]. The MRT sensor operates at a fixed frequency determined by the microwave-inducing resonator's resonance wavelength. The sensor's design structure affects its resonance frequency behavior. The resonator accumulates additional electric field energy during material interaction, which results in a frequency shift towards lower values [27]. The combination of microwave resonance technology provides an effective solution for grain moisture content measurement through shifts in resonance frequency and changes in bandwidth. Water affects microwave signals through modifications in dielectric properties which both modify resonance frequency and enlarge bandwidth. The increase in the moisture content of the grains leads to a decrease in the resonance frequency but to a widening of the bandwidth. These alterations improve the precision of moisture detection [48].

**Fig 3:**
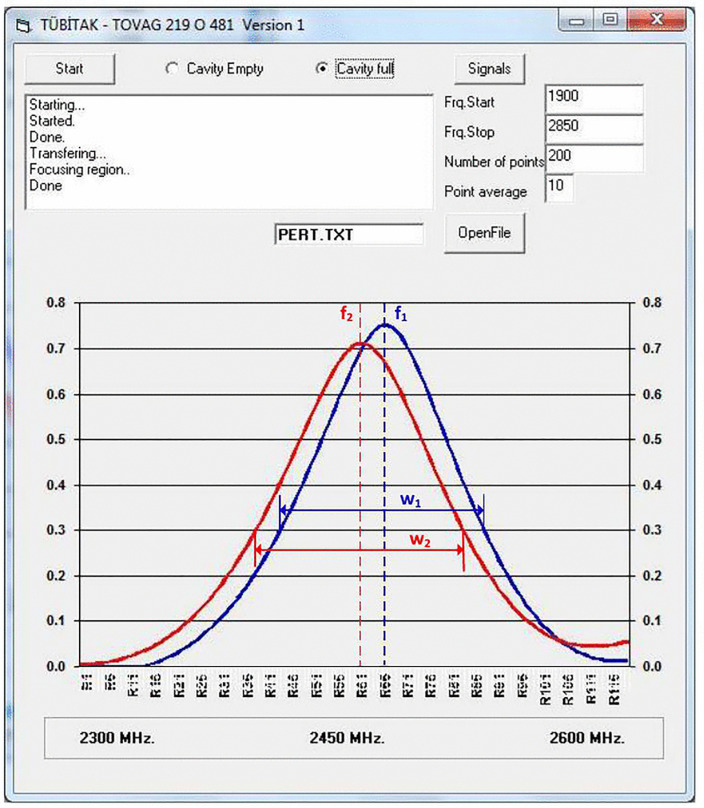
Microwave resonance curves.


The MRT system developed for measurement produces two key values, including resonance frequency shift (Δ*f*) and bandwidth increase (Δ*B*) (Fig 3). Both parameters were influenced by the moisture content and bulk density of the material under test. The ratio of the bandwidth increase (Δ*B*) to the resonant frequency shift (Δ*f*) provides density-independed [49] moisture results since it removes the bulk density effects [28]. Since the amount of water in the material does not change, the ratio of these two parameters provides a moisture measurement independent of the physical properties [27]. In microwave resonance perturbation measurements, the moisture content function, *M*(*Ψ*), can be expressed as follows [49]:

$$M(𝛹)=\frac{\Delta B}{\Delta f}=\frac{\left(w_{\text{2}}-w_{\text{1}}\right)}{\left(f_{\text{1}}-f_{\text{2}}\right)}$$

where Δ*B* is the increase in the half-width in Hz, Δ*f* is the shift in the resonance frequency in Hz, *f*_1_ is the resonance frequency of the empty resonator in Hz, *f*_2_ is the resonance frequency of the filled resonator in Hz, *w*_1_ is the half-width of the resonance of the empty resonator in Hz, and *w*_2_ is the half-width of the resonance of the filled resonator in Hz. The ratio of these quantities remains independent of changes in mass and bulk density and depends solely on the moisture content [29]. The obtained moisture content function, *M*(*Ψ*), is related to the material's relative moisture content. The MC of wheat and chickpea samples was determined by calibrating the product moisture content against *M*(*Ψ*).

## 3. Results

### 3.1. Performance results of the moisture content estimation

**Fig 4:**
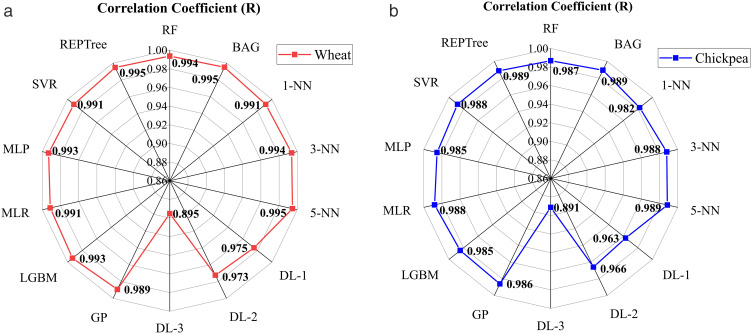
The correlation coefficient results of M(Ψ) and moisture content and on the radarmap of the artificial intelligence models. (A) Wheat, (B) Chickpea.

In Fig 4A, the MC of wheat was estimated from the moisture content function *M*(*Ψ*), and the correlation coefficients obtained are shown. For moisture prediction, the highest R was 0.995 in the BAG, 5-NN, and REPTree models. This shows that the models successfully trained the dataset and achieved strong predictive performance. RF and 3-NN models had R values of 0.994, and LGBM and MLP models had R values of 0.993. Here, it is shown that the models exhibit very effective generalization performance. The SVR, MLR, and 1-NN models also yielded balanced and successful results, with a correlation coefficient of 0.991. On the other hand, the DL-3 model performed the worst among the models, with an R value of 0.895. In addition, the DL-1 and DL-2 models had moderate results with correlation coefficients of 0.975 and 0.973, respectively. In general, the tree and k-NN models, as well as the bagging model, performed effectively for wheat moisture estimation. The DL-3 model did not produce the desired results.

Fig 4B shows the correlation coefficients obtained by estimating the MC of chickpeas from the moisture content function *M*(*Ψ*). The highest R values were 0.989 for the BAG, 5-NN, and REPTree models. These models were followed by the 3-NN, MLR, and SVR models with R values of 0.988 and the RF model with R values of 0.987. However, the DL-3 model showed the lowest performance with an R value of 0.891. The DL-1 (0.963) and DL-2 (0.966) models also showed lower R values compared to other models. LightGBM, a robust model, showed balanced, high performance with an R value of 0.986. In general, BAG, 5-NN, and REPTree were the most effective models for moisture prediction in chickpeas. Deep learning models, on the other hand, generally remained behind the other models in moisture prediction.

Table 1 presents the MAPE, MAE, and RMSE results for moisture content estimation in wheat and chickpea. Among the mean absolute percentage error values of the models used in the data sets, the lowest results for wheat were obtained with the LGBM (0.0079%) and BAG (0.0088%) models, whereas the highest results were obtained with the GP (0.1281%) and DL-1 (0.1169%) models. For chickpeas, the models with the highest MAPE results were LGBM (0.0190%) and BAG (0.0198%), whereas the highest values were found in the GP (0.1350%) and DL-1 (0.1169%) models. In addition, the REP Tree, SVR, MLP, MLR, k-NN, DL-3, DL-2, and RF models exhibited moderate error values.

**Table 1:** The MAPE, RMSE, and MAE mean and standart deviation results of the artificial intelligence models.

Model	MAPE (%)		RMSE (%)		MAE (%)	
	Wheat	Chickpea	Wheat	Chickpea	Wheat	Chickpea
RF	0.0092 ± 0.0017	0.0202 ± 0.0020	0.2349 ± 0.0319	0.3355 ± 0.0179	0.1218 ± 0.0228	0.2448 ± 0.0176
BAG	0.0088 ± 0.0016	0.0198 ± 0.0015	0.2070 ± 0.0288	0.3015 ± 0.0141	0.1175 ± 0.0211	0.2427 ± 0.0137
1-NN	0.0090 ± 0.0020	0.0203 ± 0.0022	0.2728 ± 0.0337	0.3888 ± 0.0221	0.1200 ± 0.0279	0.2440 ± 0.0215
3-NN	0.0094 ± 0.0019	0.0201 ± 0.0018	0.2304 ± 0.0354	0.3203 ± 0.0146	0.1240 ± 0.0250	0.2457 ± 0.0158
5-NN	0.0093 ± 0.0019	0.0204 ± 0.0015	0.2129 ± 0.0318	0.3102 ± 0.0105	0.1226 ± 0.0247	0.2506 ± 0.0137
DL-1	0.1104 ± 0.0165	0.1169 ± 0.0186	1.6279 ± 0.1730	1.6198 ± 0.1791	1.3068 ± 0.1611	1.2937 ± 0.1704
DL-2	0.0368 ± 0.0046	0.0427 ± 0.0049	0.6325 ± 0.0531	0.6589 ± 0.0466	0.4737 ± 0.0568	0.5173 ± 0.0487
DL-3	0.0908 ± 0.0182	0.0980 ± 0.0202	1.4851 ± 0.1996	1.4869 ± 0.2010	1.0256 ± 0.1874	1.0371 ± 0.1962
GP	0.1281 ± 0.0163	0.1350 ± 0.0184	1.8333 ± 0.1595	1.8190 ± 0.1709	1.5457 ± 0.1541	1.5207 ± 0.1634
LGBM	0.0079 ± 0.0014	0.0190 ± 0.0020	0.2423 ± 0.0223	0.3675 ± 0.0206	0.1056 ± 0.0194	0.2344 ± 0.0210
MLR	0.0165 ± 0.0007	0.0215 ± 0.0024	0.2729 ± 0.0195	0.3190 ± 0.0295	0.2173 ± 0.0155	0.2659 ± 0.0262
MLP	0.0151 ± 0.0023	0.0233 ± 0.0051	0.2467 ± 0.0400	0.3539 ± 0.0563	0.1992 ± 0.0339	0.2866 ± 0.0514
SVR	0.0164 ± 0.0009	0.0214 ± 0.0025	0.2745 ± 0.0227	0.3210 ± 0.0314	0.2175 ± 0.0180	0.2653 ± 0.0280
REPTree	0.0089 ± 0.0017	0.0203 ± 0.0012	0.2109 ± 0.0262	0.3117 ± 0.0132	0.1183 ± 0.0235	0.2492 ± 0.0012

In this study, the lowest MAE values were obtained with the LGBM and BAG models: 0.1056% and 0.1175% for wheat, and 0.234%4 and 0.2427% for chickpea. These MAE results indicate that the prediction accuracies of these models are quite high. However, the GP and DL-1 models had the highest MAE values with 1.5457% and 1.3068% for wheat and 1.5207% and 1.2973% for chickpea. In addition, the RF, MLR, MLP, SVR, and REP Tree models provided reasonable performances. Although the error values of these models were relatively low, they remained behind those of the best models. In general, the LGBM and BAG models provided the lowest MAE values for both datasets (Table 1).

The lowest RMSE values were obtained for the BAG (0.2070%), REPTree (0.2109%), and 5-NN (0.2129%) models for wheat. On the other hand, the highest RMSE values were found for GP, DL-1, and DL-2 with 1.8333%, 1.6279%, and 1.4851%, respectively. For chickpeas, the BAG, 5-NN, and REPTree models had the lowest RMSEs of 0.3015%, 0.3102%, and 0.3117%, respectively. However, the GP, DL-1, and DL-2 models had the highest RMSEs of 1.8190%, 1.6198%, and 1.4869%, respectively. This showed that the DL models were insufficient for learning the relationships in the data set. In general, the RF, BAG, and k-NN models were found to be the most effective for both datasets. Although these models had acceptable error rates, they were higher than those of other models. In general, the LGBM and BAG models were the most effective, achieving the lowest error rates across both datasets.

**Fig 5:**
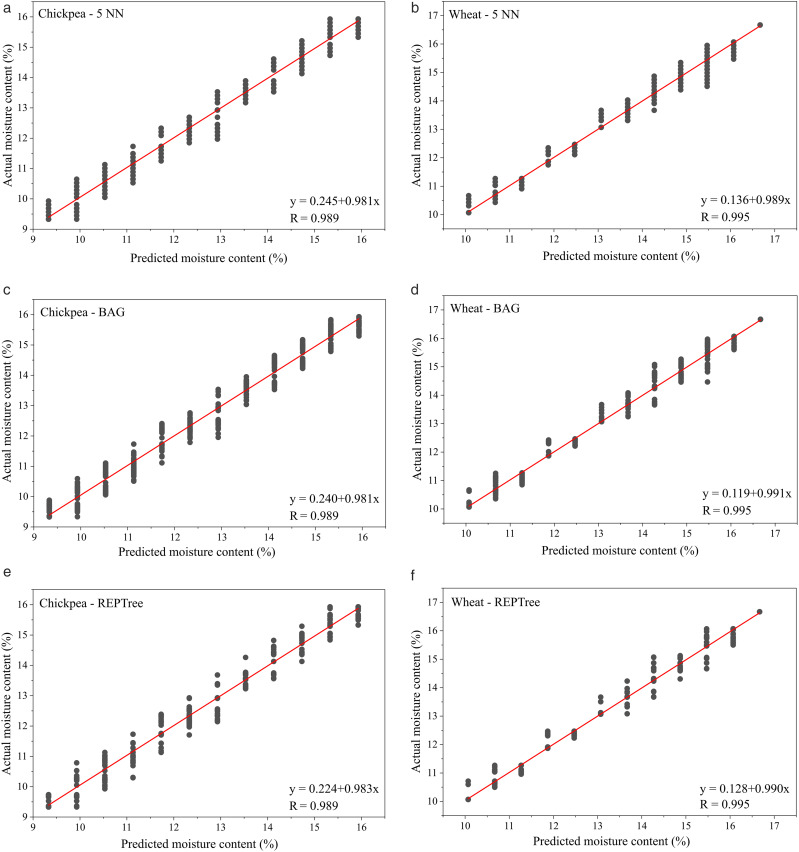
Model equations, correlation coefficients and scatter plots, and of the best models for moisture content. (A) Chickpea 5NN, (B) Wheat 5NN, (C) Chickpea BAG, (D) Wheat BAG, (E) Chickpea REPTree, (F) Wheat REPTree.

Generated equations and scatter plots for the best models (5NN, BAG, and REPTree) in the moisture content estimation are given in Fig 5. The scatter plot and model equation obtained using the 5NN model for the chickpea are presented in Fig 5A. The 5NN model showed a very strong correlation (R = 0.989) with the actual moisture content values. The regression equation (y = 0.245 + 0.981x) reveals a slope very close to 1, indicating minimal deviation from ideal predictions and high accuracy in modeling moisture content. As seen in the graph, the tight clustering of data points around the regression line throughout the entire data range confirms the high accuracy and robustness of the 5NN model for chickpeas. The scatter plot and regression equation obtained using the 5NN algorithm for the wheat dataset are presented in Fig 5B. The 5NN model achieved a near-perfect correlation (R = 0.995) with the actual moisture content values. The obtained regression equation (y = 0.136 + 0.989x) has a coefficient very close to the ideal prediction slope (1), indicating that the model's systematic deviation is negligible and that the prediction accuracy is high. The data points exhibit very tight clustering along the regression line, with minimal dispersion. These findings confirm that the 5NN model performs better and is more stable than other models in predicting wheat moisture content.

The estimation results and scatter plot obtained using the BAG model for the chickpea dataset are shown in Fig 5C. The BAG model demonstrated a strong relationship, achieving a very high correlation coefficient (R = 0.989) with the actual moisture content values. The obtained regression equation (y = 0.240 + 0.981x) yields a value very close to the ideal prediction slope of 1, indicating that the model is largely free of systematic errors and predicts moisture content with high accuracy. The tight clustering and narrow distribution of data points around the regression line in the graph demonstrate that the BAG model exhibits robust, reliable performance in predicting chickpea moisture content. The scatter plot and model equation showing the performance of the BAG model on the wheat dataset are presented in Fig 5D. The BAG model demonstrated superior predictive performance, achieving a near-perfect correlation coefficient of R = 0.995 between actual and predicted moisture values. The obtained regression equation (y = 0.119 + 0.991x) has a coefficient close to the ideal slope of 1; this indicates that the model's systematic deviation is negligible and that the predictions closely match the actual values. The tight clustering of data points along the regression line in the graph, along with the extremely low dispersion, confirms that the BAG model has high accuracy and robustness in predicting wheat moisture content.

The estimation performance and scatter plot of the REPTree model for the chickpea dataset are shown in Fig 5E. The REPTree model demonstrated strong predictive capability, with a high correlation (R = 0.989) with actual moisture content values. The obtained regression equation (y = 0.224 + 0.983x) provides a coefficient very close to the ideal slope value (1), indicating that the model's systematic deviation is minimal and that the predictions are in high agreement with the actual values. The data points exhibit tight clustering within a narrow band around the regression line, indicating that the REPTree model achieves high accuracy and robustness in predicting chickpea moisture content. The modeling results and scatter plot obtained using the REPTree algorithm for the wheat dataset are shown in Fig 5F. The REPTree model demonstrated superior performance, achieving a near-perfect correlation coefficient (R = 0.995) between actual and predicted moisture values. The model's regression equation (y = 0.128 + 0.990x) yields a coefficient extremely close to the ideal slope of 1, indicating that the model shows no systematic deviation and that the predictions match the actual values almost perfectly. The tight clustering of the data points along the regression line in the graph and the minimal dispersion confirm that the REPTree model has high accuracy and robustness in predicting wheat moisture content.

### 3.2. Classification of the wheat and chickpea

**Fig 6:**
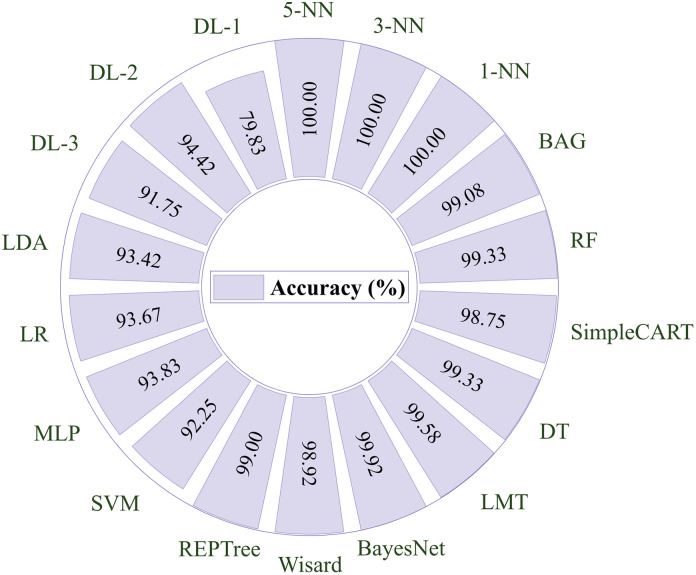
Classification accuracy results for artificial intelligence models.

The accuracy results obtained from the classification of wheat and chickpea by MC and the moisture content function *M*(*Ψ*) are shown in Fig 6. In this study, 1-NN, 3-NN, and 5-NN models exhibited the best high performance with a 100% accuracy rate. This showed that the models were fully adapted to the training set and made all predictions correctly. These models were followed by BayesNet and LMT, achieving 99.92% and 99.58% accuracy, respectively. Again, two models, RF and DT, with 99.33% and 99.33% accuracy, respectively, explained a successful result. These models performed well and captured the structure of the data set well. On the other hand, the DL-1 model had the lowest accuracy with 79.83%. In addition, the closest model to this model was DL-3 (91.75%). This showed that 17 models had a classification accuracy above 91.75%. In conclusion, k-NN models demonstrated high classification accuracy.

**Fig 7:**
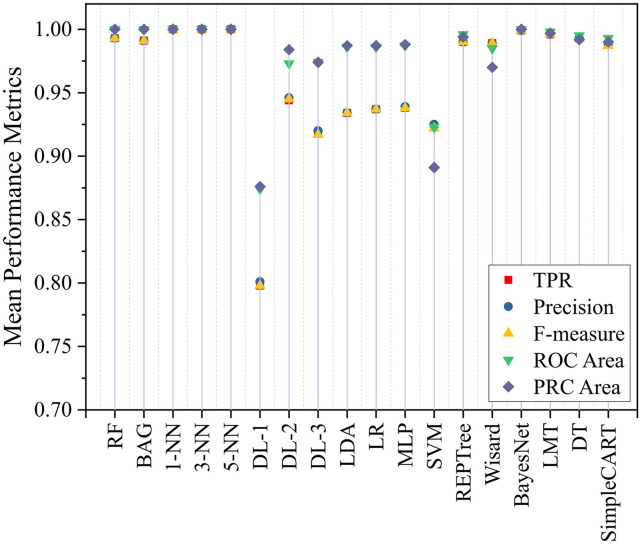
Mean performance metrics of classification for themodel.

The performance metrics and confusion matrix for wheat and chickpea are given in Table 2. The TPR, precision, f-measure, ROC area, and PRC area performance metrics of the models are shown in Fig 7. The highest TPR, precision, f-measure, ROC area, and PRC area values were 1.000 for the k-NN models. In addition, the RF, BAG, and BayesNet models also reached 1.000 for the ROC and PRC area values. The aforementioned models achieved high accuracy and correctly separated true positives. In addition, the high ROC and PRC areas showed that their classification and generalization capabilities were quite strong. The REPTree, Wisard, LMT, and DT models had moderate success. In these models, the TPR, precision, and f-measure values were above 0.989. In addition, these models had ROC and PRC areas above 0.970. These findings indicated that the models exhibited balanced and acceptable performance. On the other hand, the TPR, precision, and F-measure values of the deep learning models (DL-1, DL-2, and DL-3) varied between 0.798 and 0.945, and the ROC and PRC area values varied between 0.874 and 0.984 (Table 1A). These results were poor in terms of positive line recognition and overall accuracy. As a result, the RF, BAG, BayesNet, and k-NN models achieved the best results across all metrics and stood out for classification performance. DL models, however, require improvements due to poor performance. In addition, the remaining models were within acceptable ranges with a balanced performance.

**Table 2:** The performance metrics and confusion matrix for wheat and chickpea.

Models	Predicted		Actual Class	TPR	P	F	ROC	PRC
RF	*Wheat*	*Chickpea*						
	599	1	*Wheat*	0.998	0.988	0.993	1.000	1.000
	7	593	*Chickpea*	0.988	0.998	0.993	1.000	1.000
BAG	*Wheat*	*Chickpea*						
	599	1	*Wheat*	0.998	0.984	0.991	1.000	1.000
	10	590	*Chickpea*	0.983	0.998	0.991	1.000	1.000
1-NN	*Wheat*	*Chickpea*						
	600	0	*Wheat*	1.000	1.000	1.000	1.000	1.000
	0	600	*Chickpea*	1.000	1.000	1.000	1.000	1.000
3-NN	*Wheat*	*Chickpea*						
	600	0	*Wheat*	1.000	1.000	1.000	1.000	1.000
	0	600	*Chickpea*	1.000	1.000	1.000	1.000	1.000
5-NN	*Wheat*	*Chickpea*						
	600	0	*Wheat*	1.000	1.000	1.000	1.000	1.000
	0	600	*Chickpea*	1.000	1.000	1.000	1.000	1.000
DL-1	*Wheat*	*Chickpea*						
	451	149	*Wheat*	0.752	0.829	0.788	0.874	0.897
	93	507	*Chickpea*	0.845	0.773	0.807	0.874	0.855
DL-2	*Wheat*	*Chickpea*						
	512	88	*Wheat*	0.854	0.807	0.830	0.973	0.932
	79	521	*Chickpea*	0.961	0.972	0.967	0.973	0.994
DL-3	*Wheat*	*Chickpea*						
	527	73	*Wheat*	0.878	0.953	0.914	0.974	0.979
	26	574	*Chickpea*	0.957	0.887	0.921	0.974	0.970
LDA	*Wheat*	*Chickpea*						
	554	46	*Wheat*	0.923	0.944	0.933	0.986	0.988
	33	567	*Chickpea*	0.945	0.925	0.935	0.986	0.986
LR	*Wheat*	*Chickpea*						
	560	40	*Wheat*	0.933	0.940	0.936	0.986	0.988
	36	564	*Chickpea*	0.940	0.934	0.937	0.986	0.986
MLP	*Wheat*	*Chickpea*						
	551	49	*Wheat*	0.918	0.957	0.937	0.987	0.988
	25	575	*Chickpea*	0.958	0.921	0.940	0.987	0.987
SVM	*Wheat*	*Chickpea*						
	529	71	*Wheat*	0.882	0.960	0.919	0.923	0.906
	22	578	*Chickpea*	0.963	0.891	0.926	0.923	0.876
REPTree	*Wheat*	*Chickpea*						
	598	2	*Wheat*	0.997	0.984	0.990	0.996	0.993
	10	590	*Chickpea*	0.983	0.997	0.990	0.996	0.996
Wisard	*Wheat*	*Chickpea*						
	600	0	*Wheat*	1.000	0.979	0.989	0.989	0.978
	13	587	*Chickpea*	0.978	1.000	0.989	0.980	0.962
BayesNet	*Wheat*	*Chickpea*						
	600	0	*Wheat*	1.000	0.998	0.999	1.000	1.000
	1	599	*Chickpea*	0.998	1.000	0.999	1.000	1.000
LMT	*Wheat*	*Chickpea*						
	600	0	*Wheat*	1.000	0.992	0.996	0.998	0.996
	5	595	*Chickpea*	0.992	1.000	0.996	0.999	0.999
DT	*Wheat*	*Chickpea*						
	599	1	*Wheat*	0.998	0.988	0.993	0.995	0.991
	7	593	*Chickpea*	0.988	0.998	0.993	0.995	0.994
SimpleCART	*Wheat*	*Chickpea*						
	595	5	*Wheat*	0.992	0.983	0.988	0.993	0.987
	10	590	*Chickpea*	0.983	0.992	0.987	0.993	0.993

## 4. Discussion

Multiple machine learning (ML) techniques were employed in this research to forecast wheat and chickpea seed moisture levels, and their predictive outcomes were analysed in detail. Drying granular agricultural products after harvest is essential to maintaining their moisture content (MC) below a certain threshold, ensuring safe storage before they are made available to consumers. During this process, accurate MC measurement is crucial, as under-drying can lead to product deterioration, while over-drying results in high energy costs. Rather than applying machine learning in a black-box manner, we embedded AI modeling within the physical principles of dielectric measurement. The M(Ψ) function, derived from resonance shifts and bandwidth, carries measurement-specific significance, allowing the models to interpret data not as arbitrary inputs, but as physically meaningful signals. This ensures that AI is used not in isolation but as a measurement-enhancing tool.

Electronic moisture meters, which are frequently used to determine the MC of grains, provide an average measurement for a sample (usually over 10 g). This can lead to erroneous results, particularly if the sample contains kernels with significantly varying MC levels. In practice, MC is determined by averaging the results of three or more measurement trials, which may not reliably reflect the MC distribution within the sample. Identifying whether MC levels are uniformly distributed or if the kernels exhibit high variance in their physical properties is beneficial for optimising drying, storage, and transportation processes. Consequently, estimating MC at the kernel level or in very low-mass samples is essential, which necessitates the design of sensitive, non-invasive, and low-cost moisture meters.

In this study, a microwave dielectric spectroscopy setup based on a perturbed cavity resonator was designed. The acquired data were then processed using different ML algorithms to estimate the MC of agricultural products. The system enables MC measurement independently of physical characteristics such as size, volume, or sphericity, making it suitable for both granular products and kernel-shaped foods such as legumes. Experiments were conducted on wheat and chickpea kernels, which represent the former and latter categories, respectively. To standardise measurements, samples were placed in the cavity either by a fixed number of kernels (three or four chickpeas) or by a fixed mass (6–10 g for wheat). For each MC level, 50 trials were performed to minimise measurement errors, which were demonstrated to remain below 1%.

The cavity perturbation method was selected for its high sensitivity, enabling the detection of small changes in MC. Compared to other microwave characterisation techniques, such as open-ended coaxial cable or free-space methods, cavity perturbation offers superior performance due to minimal energy loss in the confined system, resulting in a high-quality factor. The main advantage of the proposed system is that it replaces an expensive vector network analyser with a custom-integrated microwave setup comprising various RF components, thereby achieving a similar spectroscopy system at a significantly lower cost. VNAs are costly instruments, restricting the operation of many state-of-the-art microwave-based characterisation systems to laboratory environments. However, the system presented in this work employs an RF geometry that not only enables VNA operation but also digitally processes signals and communicates with a PC to convey data.

To ensure statistical robustness and mitigate model bias, a strictly balanced dataset design was employed. Exactly 50 measurements were acquired for each of the 12 distinct moisture levels, resulting in a uniform class distribution of 600 samples per grain type. This balance prevents the machine learning models from becoming biased towards specific moisture ranges. Regarding preprocessing, three steps (physical preprocessing, signal preprocessing, and feature extraction) were implemented: (I) Manual screening was performed to remove broken or immature seeds. (II) The raw I/Q signals were smoothed using the hardware’s synchronous digital filter. (III) The moisture function M(*Ψ*) was calculated as a ratio of bandwidth to frequency shift. This calculation serves as a critical normalization step, effectively removing the variance caused by bulk density and sample volume before the data is fed into the ML algorithms.

This study combines a highly sensitive microwave characterisation technique with ML methods to predict MC in two essential agricultural grain types. The demonstrated measurement technique and setup can also be extended to MC determination for other agricultural products and artificial materials in granular form. Although cavity perturbation has been widely used for the dielectric characterisation of agricultural products, to the authors’ knowledge, no previous studies have applied ML methods to MC estimation. The most recent work in this area [33] employed open-ended coaxial probe techniques and cavity perturbation simultaneously on various food products, estimating MC by tracking changes in complex permittivity at 915 MHz and 2.5 GHz.

The vast majority of recent literature on microwave-based methods for MC measurement of grains employs free-space techniques [16,50–55]. Despite allowing telemetric measurements, as previously mentioned, these techniques are not as highly sensitive as the resonant-based microwave methods. On the other hand, resonance-based methods, including the cavity perturbation technique, are only suitable for laboratory characterisation and not the best option for applications requiring in situ measurements, e.g., wireless measurement of grains stored in a silo. Regardless, a high sensitivity, good resolution, and reliable theoretical background are among the appealing features of the resonant-based techniques. In addition, it demonstrates a custom microwave dielectric measurement setup that replaces the VNA. To the best of our knowledge, this is the first study among cavity resonator-based methods to do so, and one of the few among all microwave-based methods, the others being [56] and [16]. In [56], a handheld device based on a double-ring sensing probe geometry was demonstrated for measuring the MC of corn ears. Nevertheless, this work is aimed towards the measurement of the MC of a single corn ear and cannot be adapted to the measurement of agricultural products in granular form since the operation frequency and the calibration parameters are all optimized specifically for corn ears. In [16], a free-space method based on traveling–standing wave attenuation variation with sample MC was demonstrated to measure the MC of rice and corn samples. There are two main differences between this work and the work presented in this paper: 1) In [16], the frequency is fixed and cannot be swept, whereas our setup can be set to perform a frequency sweep between 500 MHz to 4 GHz in 1 MHz steps (corresponding to 3501 measurement points), 2) The measurements in [16] are scalar; meaning only attenuation of the signal is measured, whereas the measurements in this work has both the phase and magnitude information for all frequency samples within the spectrum. In that sense, the work presented is a low-cost replica of a VNA, with particular focus on MC and bulk density measurements. These differences constitute a superiority in this work’s favor, since measurements can be repeated at a desired frequency only by changing the input parameters, and both attenuation and phase shift information can be used to estimate MC and bulk density.

ML techniques demonstrated superior performance in predicting seed MC compared to traditional methods, offering enhanced accuracy, efficiency, and scalability [57]. ML models outperform conventional techniques because they can identify and analyse complex non-linear relationships between input variables and MC, which traditional methods struggle to handle effectively [58]. A key advantage of ML models is their capacity to process large datasets by integrating environmental data and soil characteristics with remote sensing elements [59], resulting in more precise predictions. Model performance improves further when optimised through hyperparameter tuning and feature selection.

However, ML models present certain challenges. Their implementation requires large amounts of high-quality training data, which may be difficult to obtain. Additionally, complex ML models can be difficult to interpret, raising concerns about their reliability in practical applications [60]. Their performance heavily depends on the quality and representativeness of the training data, which affects their ability to generalise to new conditions. While ML techniques offer significant improvements in seed MC prediction, their adoption should be approached with caution due to data limitations, interpretability, and generalisation issues. Integrating ML with physical models and explainable AI methods can help overcome these limitations while enhancing prediction accuracy and reliability [61].

Microwave resonance technology provides moisture data independent of material density; however, certain physical and environmental limitations can affect the system's accuracy. In particular, the shape and geometry of the object significantly influence measurement precision. While spheres like soybeans yield high accuracy, the variability in shape among irregular grains such as wheat or corn increases measurement uncertainty [62]. Grain shape and sample volume can induce non-linearities, while modest errors may also result from external electromagnetic interference and calibration drift in the microwave resonant measuring equipment. Despite the cavity's metal casing providing protection against external interference, it remains crucial to ensure the internal RF connections are secure to prevent undesired signals that may disrupt the resonance profile. This study mitigated these effects through shielding and meticulous measurement conditions; however, prolonged or extensive use may result in gradual calibration alterations beyond mere temperature influences, potentially due to aging semiconductor components or moisture from residual grain deposits. Therefore, it is advisable to routinely verify the zero point to maintain measurement accuracy in practical applications. Furthermore, in open sensor designs (stray field resonators), radiation losses caused by the dispersion of electromagnetic energy into the environment can lead to artificial broadening of the bandwidth, which may be mistaken for the dielectric losses of the material and result in measurement errors. Another key limitation of the system is thermal stability; temperature fluctuations during operation cause thermal expansion of the resonator, leading to calibration drift in the resonance frequency. Without effective temperature compensation, these drifts can lead to significant errors in moisture calculations [63]. Furthermore, exceeding certain moisture content levels causes the sensor response to become non-linear, limiting the measurement range of single-resonator systems in particular [29,64]. These factors require advanced calibration algorithms and the use of temperature-resistant materials to ensure the system's industrial reliability [63].

Regarding the generalization capability and uncertainty of the proposed system, it is important to note that the dielectric properties of agricultural products are inherently dependent on their biochemical composition and structural characteristics. Although the sensor hardware and the derived physical metric M(Ψ) are universal and density-independent, the machine learning models established in this study are specific to the dielectric signature of wheat and chickpea. Consequently, applying this sensor to other grain types would require retraining the models with data specific to those new materials to capture their unique moisture-dielectric relationships. However, the use of 10-fold cross-validation in this study, which tests the model on unseen subsets of data in each iteration, provides a robust estimation of the model's performance and confirms that the system can reliably predict moisture content for the trained grain types without overfitting.

ML-based moisture prediction techniques offer various advantages over conventional methods, particularly in terms of speed, non-destructive analysis, and cost-efficiency. The analysis included RF, BAG, k-NN, and SVR, all of which produced promising results. Among these, BAG and k-NN exhibited the strongest correlations, as indicated by their high correlation coefficients. However, when applied, DL models achieved lower success rates. The primary strength of ML models lies in their ability to process large datasets while simultaneously assessing the effects of various parameters. However, limitations include the need for extensive data and optimised model parameters. Some ML algorithms also demand higher computational power than conventional techniques.

## 5. Conclusion

This study presents an innovative, low-cost microwave resonance perturbation measurement system capable of non-destructive sensing of the moisture content (MC) of wheat and chickpea grains. Machine learning was not used as a generic predictive tool but as a physically grounded modeling strategy that enhances the precision of microwave dielectric measurements in a cost-effective and non-invasive manner. One important way to obtain precise, fast moisture estimation is to integrate microwave resonance technology with machine learning (ML) models, an alternative to traditional moisture measurement methods. The main findings of this research can be reworded as follows:

- The developed microwave resonator sensor was able to predict the moisture content with high accuracy for both wheat and chickpea, as evidenced by the correlation coefficient of the best-performing models, which was 0.995 for wheat and 0.989 for chickpea.
- Among the tested ML models, BAG, k-NN, and REPTree performed better than the deep learning models.
- In addition, the lowest RMSE values for wheat were derived using BAG (0.2070), REPTree (0.2109), and 5-NN (0.2129), whereas the lowest RMSE values for chickpea were produced with BAG (0.3015), 5-NN (0.3102), and REPTree (0.3117).
- The 1-NN, 3-NN, and 5-NN models in the classification using the moisture function M(Ψ) exhibited excellent classification performance of 100%.

The proposed system is compared to conventional methods and provides a non-destructive, rapid, and low-cost alternative, and it does not require an expensive vector network analyzer. In this study, we used the resonance perturbation technique, which offers high sensitivity, low energy loss, and good accuracy for microwave magnetometers. Since the environmental effects could be minimized with the use of a closed resonator structure rather than open-ended coaxial probes or free space transmission methods, more precise dielectric characterization was possible. This study addresses a clear measurement problem nondestructive moisture estimation using a custom-built cavity perturbation sensor. The proposed metric and ML integration offer a scalable, cost-effective alternative to traditional VNA systems. The methodology is rooted in measurement science, not merely data analytics. It could also serve as a basis for future work to improve and adapt the system to other agricultural applications. The integration of IoT and Edge AI enables grain storage and processing processes to be more efficient by providing real time moisture monitoring. In addition, it can be adapted to other grains, such as corn, sorghum, lentils, barley, and rice, providing a wider range of applications. Optimizing the machine learning model will improve the prediction accuracy. A compact, portable instrument design would enable practical field use, providing a fast, accurate, and cost-effective solution. The resulting improvements will enable more affordable, smart agricultural applications and more affordable moisture estimation.

The system's low-cost radio frequency architecture and design, which obviates reliance on a VNA, establishes a robust basis for the device's mass production and commercial prototyping. The suggested perturbation system serves as a portable, on-site moisture meter, suitable for integration into grain processing activities for quick, nondestructive monitoring. Subsequent development phases will emphasize thermal stability and calibration resilience in extreme environmental circumstances to achieve industrial certification criteria. The device's compact dimensions and compatibility with Edge AI and Internet of Things systems offer opportunities to market it as an economical quality-control solution that integrates seamlessly with existing grain-processing systems. These qualities will enable dependable utilization in conditions of real-world variability.

### Nomenclature

**Table:** 

*ADC*	Analog-to-digital converter
*BAG*	Bagging
*BayesNet*	Bayesian network
*ANN*	Artificial neural network
*DAC*	Digital-to-analog converter
*DL*	Deep learning
*DSP*	Digital signal processing
*DT*	Decision tree
*f*_1_	Resonance frequency of the empty resonator, Hz
*f*_2_	Resonance frequency of the filled resonator, Hz
*GP*	Gaussian processes
*H*	Mean relative error of calibration
*I/Q*	In-phase and quadrature
*kNN*	k-nearest neighbors
*LDA*	Linear discriminant analysis
*LightGBM*	Light gradient boosting machine
*LMT*	Logistic model tree
*LPF*	Low-pass filter
*LR*	Logistic regression
*MAE*	Mean absolute error
*MAPE*	Mean absolute percentage error
*MC*	Moisture content, % w.b.
*M*(*Ψ*)	Moisture content function
*MCU*	Microcontroller unit
*ML*	Machine learning
*MLP*	Multilayer perceptron
*MLR*	Multiple linear regression
*MRT*	Microwave resonance technology
*PLL*	Phase-locked loop
*Q*	Cavity quality factor
*Q*_*1*_	Quality factors for the empty cavity
*Q*_*2*_	Quality factors for the filled cavity
*R*	Correlation coefficient
*REPTree*	Reduced-error pruning tree
*RF*	Random forest
*SEC*	Standard error of calibration
*SimpleCART*	Simple classification and regression tree
*SMA*	SubMiniature version A
*SSC*	Soluble solid content, /Brix
*SVM*	Support vector machine
*SVR*	Support vector regression
*S*_21_	Magnitude of the complex transmission parameter
*VCO*	Voltage-controlled oscillator
*VGA*	Variable-gain amplifiers
*VNA*	Vector network analyzer
*Wisard*	Wilkie, Stonham and Aleksander recognition device
*w*_1_	Half-width of the resonance of the empty resonator, Hz,
*w*_2_	Half-width of the resonance of the filled resonator, Hz
*ε* ^*^	Complex relative permittivity
Δ*B*	Increase of the half-width, Hz
Δ*f*	Shift in the resonance frequency, Hz

## Supporting information

S1 File Inclusivity in global research questionnaire. (DOCX)
